# Increased Serum Levels of Activated Caspases in Murine and Human Biliary Atresia

**DOI:** 10.3390/jcm10122718

**Published:** 2021-06-19

**Authors:** Omid Madadi-Sanjani, Gunnar Bohlen, Fabian Wehrmann, Julia Andruszkow, Karim Khelif, Reinhard von Wasielewski, Heike Bantel, Claus Petersen

**Affiliations:** 1 Department of Pediatric Surgery, Hannover Medical School, 30625 Hannover, Germany; g.bohlen@kkhohz.de (G.B.); karim.khelif@ulb.ac.be (K.K.); petersen.claus@mh-hannover.de (C.P.); 2 Department of Medicine, University of Colorado Denver, Aurora, CO 80045, USA; fabian.wermann@hotmail.de; 3 Institute of Pathology, University Hospital RWTH, 52074 Aachen, Germany; jandruszkow@ukaachen.de; 4 Department of Surgery, University Children’s Hospital Queen Fabiola, 1200 Brussels, Belgium; 5 Institute of Pathology, KRH Hannover Northern City Hospital, 30167 Hannover, Germany; reinhard.vonwasielewski@krh.de; 6 Department of Gastroenterology, Hepatology and Endocrinology, Hannover Medical School, 30625 Hannover, Germany; bantel.heike@mh-hannover.de

**Keywords:** biliary atresia, liver fibrosis, apoptosis, caspase-3 activity

## Abstract

In biliary atresia (BA), apoptosis is part of the pathomechanism, which results in progressive liver fibrosis. There is increasing evidence suggesting that apoptotic liver injury can be non-invasively detected by measuring the caspase activity in the serum. The purpose of this study was to investigate whether serological detection of caspase activation mirrors apoptotic liver injury in the infective murine BA-model and represents a suitable biomarker for BA in humans. Analysis showed increased caspase-3 activity and apoptosis in the livers of cholestatic BALB/c mice, which correlated significantly with caspase activation in the serum. We then investigated caspase activation and apoptosis in liver tissues and sera from 26 BA patients, 23 age-matched healthy and 11 cholestatic newborns, due to other hepatopathies. Compared to healthy individuals, increased caspase activation in the liver samples of BA patients was present. Moreover, caspase-3 activity was significantly higher in sera from BA infants compared to patients with other cholestatic diseases (sensitivity 85%, specificity 91%). In conclusion, caspase activation and hepatocyte apoptosis play an important role in experimental and human BA. We demonstrated that serological detection of caspase activation represents a reliable non-invasive biomarker for monitoring disease activity in neonatal cholestatic liver diseases including BA.

## 1. Introduction

During the last few decades, the overall outcome of patients with biliary atresia (BA) has been stabilized in industrial nations [1,2]. However, the survival rate with the patient’s native liver is still low and the complications and sequelae of liver transplantation (LTx), as well as the financial burden, are unsolved problems [3]. The top priority is, therefore, to unravel the etiology of BA and to turn the sequential, but only symptomatic, therapy (Kasai procedure and LTx in approximately 70–80%) into an origin-related curative and/or prophylactic approach [4,5]. Clinical research focuses on effective screening, timely and appropriate diagnosis, surgery for BA, as well as postoperative care in order to extend the survival of both native and transplanted livers [6,7,8,9]. Basic research refers either to patient specimens, which are available after the diagnosis has been confirmed, or to experimental BA, which is solely simulated in the Rhesus rotavirus (RRV) driven mouse model [10]. Under these circumstances, the translation of findings from the animal model to the human disease and to clinical application is of the highest interest [11].

In BA, an ongoing inflammatory process of the entire biliary tree leads to irreversible damage of the extrahepatic bile ducts and the deterioration of liver function. The entity is assumed to be an immunological dysregulation in genetically susceptible newborns, probably triggered by hepatotropic viruses [12]. However, the pathomechanism of the immunological process still remains unclear, although pieces of the pathophysiological jigsaw puzzle have been identified [13]. Among them, apoptosis is known to be a significant mechanism of liver injury when physiologically programmed cell death changes into a deregulated process. Apoptotic liver injury plays a role in a variety of liver diseases triggered by different signaling mechanisms, namely the extrinsic death receptor and the intrinsic mitochondrial pathway [14,15]. The extrinsic pathway is initiated by death ligand/receptor interaction, i.e., CD95L/CD95, which is followed by the activation of initiator and effector caspases. The latter, such as caspase-3 or -7, cleave a variety of cellular substrates, including cytokeratin-18 (CK-18), a major intermediate filament protein in hepatocytes [16,17]. It has been demonstrated that CD95 enhances liver injury and fibrogenesis in bile duct ligated mice, indicating that CD95 signaling plays a role in cholestatic liver injury [18]. Funaki et al. demonstrated increased apoptosis in liver biopsies of BA patients for the first time and Liu et al. showed that activation of the upstream CD95 pathway seems to be a poor prognostic factor [19,20]. In s cholestatic RRV mouse model, up-regulation of apoptosis-related genes has been shown [21,22]. Erickson et al. demonstrated that, in the experimental mouse model, apoptosis along the entire biliary tree is, together with IFNγ and TNFα, a crucial factor for developing cholestatic BA-like changes [23].

Recently, it has been shown that caspase-mediated CK-18 cleavage fragments as well as activated caspases are released from apoptotic cells and can be detected in the blood of patients with liver diseases [19,20,21]. However, whether serological detection of caspase activation might be a suitable non-invasive biomarker for the early detection of cholestatic liver diseases such as BA has, thus far, not been investigated. We therefore investigated caspase-activation in liver and sera from newborn RRV-BALB/C mice in regard to apoptotic liver injury and repeated these investigations in BA patients.

## 2. Materials and Methods

### 2.1. Experimental BA Mouse Model

BALB/c and C57BL/6 mice were purchased from Charles River Laboratory (Charles River Laboratories, Research Models and Services, Sulzfeld, Baden-Wurttemberg, Germany). The mice were kept in specific pathogen-free laminar-flow cages and subjected to a 12 h dark-light-cycle; food, water and litter were sterilized. All procedures were approved by the local animal welfare committee, in compliance with the national regulations for the protection of animals (permit number 07/1327) and performed under the supervision of a responsible veterinarian.

Intraperitoneal application of a 20-microliter saline solution containing 1 × 10^6^ pfu/mg of RRV or sterile saline in controls, was performed in all newborn mice within 24 h postpartum, as previously described [24]. Animals that died within 48 h after infection were excluded as early lethality due to technical complications. Mice were monitored daily for weight and general condition, as well as signs of cholestasis, i.e., icterus of the non-fur covered skin and acholic stools. Bilirubinuria was tested by using Bilugen-Test^TM^ (Boehringer Mannheim, Mannheim, Baden-Wurttemberg, Germany).

Sacrifice and dissection of the mice was scheduled on day 5, 8, 11 or 15 after RRV-infection and performed under a dissection microscope. Morphological changes of the gall bladder and the hepatoduodenal ligament, as well as corresponding microscopic findings in the liver, gave evidence of the experimental BA model, as previously described [25]. Explanted livers were divided and harvested for histological examination and viral mRNA determination. Small parts of the liver were fixed in formalin 4%, embedded in paraffin and stained with hematoxylin and eosin (H.E.). Remaining liver tissue samples were snap-frozen in liquid nitrogen and stored at −80 °C for future virus quantification. Up to 30 mg of liver tissue from each animal was lysed and homogenized using a TissueRuptor and RLT buffer (Qiagen GmbH, Hilden, North Rhine-Westphalia, Germany). After centrifugation for 5 min at full speed, the supernatant was collected, and a volume of 70% ethanol was added and mixed by pipetting. The sample was transferred to an RNeasy spin column and RNA was isolated following the protocol of RNeasy Mini Handbook (Qiagen GmbH, Hilden, North Rhine-Westphalia, Germany). Total RNA was quantified and validated for integrity using the U3000 (HITACHI).

### 2.2. RRV and Real-Time PCR

The virus was kindly provided by M. Riepenhoff-Talty (Buffalo, NY, USA). The Rhesus rotavirus strain (RRV, MMU 18006) was titrated in MA-104 cells and expressed as plaque forming units (pfu/mg) [24,25]. Titration of the virus in graded logarithmic dilutions was performed in tissue culture microtiter plates (Nunc, Roskilde, Denmark) with confluent cells.

Reverse Transcription and Real-Time Polymerase Chain Reaction (rt-PCR) Assay Reverse transcription was performed using Superscript III Reverse Transcriptase and random primers (Invitrogen, Karlsruhe, Germany), according to the manufacturer’s instructions. A total of 0.5 μg of RNA from each sample was reverse transcribed into single-stranded cDNA. The rt-PCR was performed using 50 ng of template for the quantitative detection of mRNA levels for the rotavirus-specific VP6 protein and glyceraldehyde-3-phosphate-dehydrogenase (GAPDH) as the internal control. We used an Mx-3000P Multiplex Quantitative PCR sequence detector (Stratagene, La Jolla, CA, USA) using SYBR Green as a double-strand DNA-specific binding dye. PCR amplifications were performed with specific primers (Supporting Information Figure S1) on a total volume of 25 μL containing 50 ng of template, 1 μL of each primer (0.35 μM), 12.5 μL of 2X Brilliant SYBR Green QPCR Master Mix (Stratagene) and 0.5 μL of a diluted reference dye (RedOrangeXanthocyanin), at a final concentration of 300 nM. An initial denaturation at 95 °C for 10 min was followed by 40 cycles (95 °C for 30 s, 51 °C for 30 s and 72 °C for 45 s). VP6 mRNA expression in the liver of RRV treated mice was normalized to the internal control and compared to a saline-treated control group. 

### 2.3. Immunohistochemical Staining

Terminal deoxynucleotidyl transferase-mediated deoxyuridine triphosphated nick end-labelling (TUNEL), as the gold standard for the detection of apoptosis, was performed together with the assessment of the cleaved caspase-3, the decisive effector enzyme in the apoptosis pathway. In brief, for TUNEL, the ApoTag In situ Apoptosis Detection Kit (CHEMICON International, Temecula CA, USA) was used for formalin-fixed, paraffin embedded (FFPE) slides of 5 µm thickness. After mounting, slides were dewaxed and permeabilized by diluted proteinase K (RT, 15′; 20 µg/mL), followed by blocking endogenous peroxidase (RT, 5′; H_2_O_2_ 3% in PBS). Sections were washed in PBS and an equilibration buffer, followed by incubation with a TdT reaction mixture (RT, 60′). After stop-wash, an anti-digoxygenin peroxidase conjugate was applied (RT; 60′), visualized by DAB substrate. Staining patterns on the slides were evaluated under a conventional light microscope. Caspase and TUNEL were assessed in each specimen by counting the immunoreactive hepatocytes, cholangiocytes and endothelial cells in 10 portal tracks with a magnification of 200. DNA fragmentation and cytoplasmic staining were considered positive for TUNEL and caspase, respectively. Inflammatory infiltrates were recorded whenever present.

Caspase-3 activity was assessed by immunohistochemistry using a polyclonal rabbit antibody (RBK009; dilution 1:200; Zytomed Systems, Berlin, Germany). The 3 µm slides were deparaffinized and a high-temperature antigen unmasking technique in a pressure cooker was applied (5′; 121 °C, 0.01 M sodium citrate buffer pH 6.0), followed by blocking, as described above. Sections were incubated overnight with a primary antibody, detection was performed by a multilink biotinylated antibody (LSAB/HRP, DAKO Glostrup, Danemark), the substrate was DAB. Negative controls were completed by omitting the primary serum that showed absence of specific staining. Adequate positive controls were included.

### 2.4. Detection of Caspase Activity in Mouse Liver Tissue

A total of 20–30 mg of liver tissue was homogenized in liquid nitrogen and lysed in 10 mM TRIS-HCl containing 0.5% Nonidet P-40, 10 mM MgCl_2_, 150 mM NaCl, 10 mM dithiothreitol and 1% protease inhibitor (Sigma-Aldrich, St. Louis, MO, USA). The liver tissue extracts were analyzed for protein concentration using a Lowry assay (BIO-RAD; Munich, Germany) and then diluted in a buffer containing 50 mM Tris-HCl (pH 7.4), 10 mM KCl and 5% glycerol for a final concentration of 1 µg/µL. Then, 15 µL extracts were incubated with 15 µL of the caspase substrate DEVD-luciferin and luciferase reagent (Caspase-Glo Promega, Mannheim, Germany) for 1.5 h at room temperature. Following cleavage of the substrate at the DEVD peptide by caspase-3 and caspase-7, aminoluciferin was released, resulting in light production in a luciferase reaction that can be measured in relative light units (RLU) using a luminometer (GloMax^®^ 96 Microplate Luminometer Mannheim, Germany). Samples were analyzed and compared to the saline treated control group [26].

### 2.5. Detection of Caspase Activity in Mouse Sera

A total of 4 µL of mouse serum was diluted in 36 µL of a buffer containing 50 mM Tris-HCl (pH 7.4), 10 mM KCl and 5% glycerol. Then, 10 µL of the dilution were incubated with 10 µL of the caspase substrate DEVD-luciferin and luciferase reagent for 3 h in the dark at room temperature. Finally, the luminescence of each sample was measured in triplicates in a luminometer (Lumat, Berthold Technologies, Bad Wildbad, Germany) and calculated as the increase relative to the saline-treated control group [26].

### 2.6. Detection of Caspase Activity in Human Sera

A total of 25 µL of human serum was diluted in 25 µL of a buffer containing 50 mM Tris-HCl (pH 7.4), 10 mM KCl and 5% glycerol. Then, 15 µL of the diluted serum was incubated with 15 µL of the caspase substrate DEVD-luciferin and luciferase reagent for 1.5 h at room temperature in triplicate. Finally, the luminescence of the samples was measured in a luminometer (GloMax^®^ 96 Microplate Luminometer, Promega, Mannheim, Germany) and calculated as relative light units [26].

### 2.7. Patients Characteristics

Sera and liver biopsies were taken from 26 BA patients (70 days old on average; range, 21–101), simultaneously with the Kasai procedure. As controls, the following two distinct groups were enrolled in the study: (i) sera from 23 age-matched healthy patients (62 days old on average; range, 2–200), who were scheduled for routine surgical procedures and (ii) sera from 11 patients with neonatal cholestasis (93 days old on average; range, 47–181). The latter showed statistically no difference to the study group in terms of gender distribution, bilirubin, AST, ALT and γGT (Supporting information Table S1). For ethical reasons, no age-matched liver biopsies were available. 

Approval was obtained from the Local Research Ethical Committee (No. 41/2006) prior to enrolling patients in this study. Patients were included in the BA research program after obtaining consent from the patient’s legal guardian.

### 2.8. Statistics

Statistical evaluation was calculated using SPSS 18 (IBM, Somers, NY, USA). A statistical analysis comparing the concentration of the M30 antigen (U/L), caspase activity (RLU) and TUNEL signals in the different mouse and patient groups at various times was performed using the unpaired two-tailed *t*-test for equality of means. The dependence between readings of M30, caspase activity, virus load and TUNEL signals was performed using Pearson’s r or, in a skewed distribution, Spearman’s rho, respectively. The difference between the occurrences of BA in both mouse strains was analyzed using the chi-square test. Values are expressed as mean ± SEM. Statistical significance was assumed for *p* < 0.05. Sensitivity and specificity were calculated by using ROC analysis.

## 3. Results

### 3.1. Induction of BA and Cholestasis in RRV-Infected Mice

The early intraperitoneal infection with RRV induces a high incidence of experimental BA in newborn BALB/c mice, while in C57BL/6-mice it induces a low incidence [24]. We pre-tested the outcome parameters of our study’s design (incidence of cholestasis and BA), at first in 192 pups (Table 1). The 192 pups were the basis of a multitude of studies from our department, using the RRV-BALB/c mice model, which is the reason for the number of animals. As previously demonstrated, we observed experimental BA in 69% of BALB/c and 22% of C57BL/6 mice (*p* < 0.001). It remains unclear thus far, whether the development of BA in this mouse model depends on the presence of the virus, or if RRV only acts as an initial trigger resulting in immune-response mechanisms that lead to bile duct destruction. Previous studies implicated that the pathomechanism, which turns cholestasis into BA, is an on-going process even after viral clearance. Therefore, we investigated the viral clearance in BALB/c pups that developed icterus after RRV-infection (*n* = 22). In line with previous studies, the virus load increased up to day eight after RRV infection, and was cleared within the next 3 days (Supporting Information Figure S2) [27].

### 3.2. Increased Caspase Activation and Apoptosis in Liver Tissues from BA Mice

**Figure 1:**
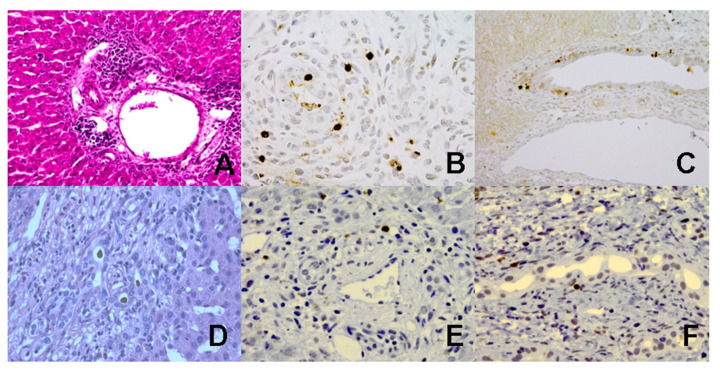
(**A**–**C**: mouse) (**A**) Representative portal tract of a liver section in HE staining, low-power. The genuine bile duct is clearly visible with perifocal mononuclear cell infiltrate. (**B**) Labeling of active caspase-3 and (**C**) TUNEL of a portal tract in high (**B**) or low power (**C**). Positively labeled cells display dark brown color signals in the nuclei. Notice that labeling is restricted to inflammatory by-standers, either perifocal or in the wall of the bile duct. Bile duct epithelium itself does not show specific signals. (**D**–**F**: human) (**D**) Advanced stage of biliary atresia with portal tract fibrosis and neo-ductuli with severe cholestasis, HE staining. (**E**) Labeling of active caspase-3 in neo-ductuli within expanded portal tracts. Staining pattern is similar to TUNEL results (**F**).

We first investigated apoptotic liver injury in cholestatic RRV+-BALB/c mice compared to control mice that received an injection of saline instead of RRV. In contrast to the saline-treated control mice, the RRV-treated BA mice showed increased TUNEL reactivity and caspase-3 activity (Figure 1A–C) in periductal regions of the liver tissue.

We then analyzed apoptotic liver injury in BALB/c mice after RRV treatment, independent from clinical cholestasis. Therefore, the virus-treated mice without clinical signs of cholestasis were defined as the second control group (*n* = 34). In contrast to the non-icteric pups, the cholestatic mice developed increased serum-bilirubin levels in the observation period of day 5–15 (Supporting Information Figure S3). 

**Figure 2:**
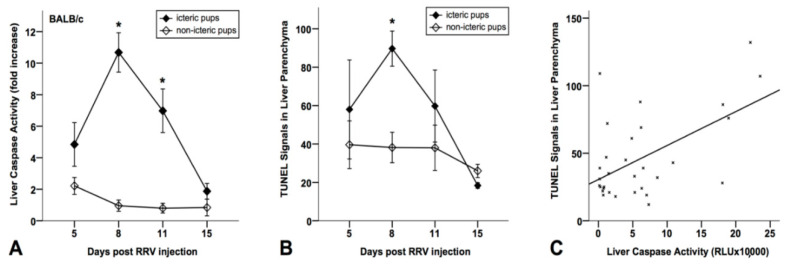
In icteric mice, caspase activity in liver tissues is significantly increased at day 5 and 8 after RRV infection (**A**). At the same time, TUNEL reactivity in liver parenchyma is elevated in both icteric and non-icteric BALB/c mice (mean ± SEM) (**B**). In icteric pups, TUNEL positivity (TUNEL-positive cells in liver parenchyma as average per portal tract) shows a peak at day 8 and decreases by the end of the second week of life. At each time point, every group contains 3 to 6 mice. Significance (*) was confirmed for day 8 (*p* = 0.005) (**A**). In 31 mice, the Pearson correlation coefficient between TUNEL-reactivity and luminometrically detected caspase-3/-7 activity in liver tissues was 0.557 (*p* = 0.001) (**C**).

As demonstrated in Figure 2A, the icteric mice (*n* = 59) showed increased caspase-3/-7 activity in the liver tissue extracts, which was significantly (*p* < 0.05) higher at day 8 and 11 compared to the non-icteric mice (*n* = 55). Similarly, during day 5–15 following the virus application, the icteric BA mice showed an increased number of TUNEL-positive cells in liver tissues (significant *p* < 0.05 at day 8) compared to the non-icteric control mice (Figure 2B). The caspase-activity significantly (*p* < 0.01) correlated (r = 0.056) with TUNEL reactivity in the liver tissues of BA mice (Figure 2C), indicating that increased caspase-activation is associated with apoptotic liver injury in those mice.

### 3.3. Increased Caspase Activity in Sera of BA Mice Compared to Non-Icteric Mice

**Figure 3:**
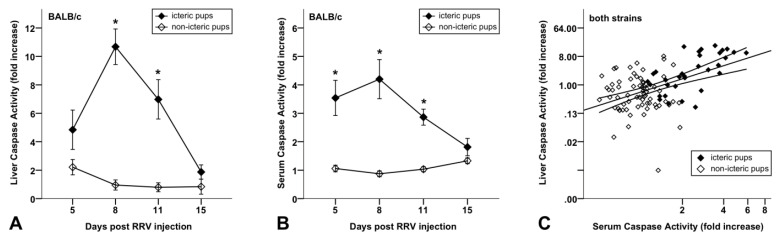
Detection of caspase-3/-7 activity measured using luminescent substrate assay (as fold increase compared to untreated controls; mean ± SEM) in liver tissues (**A**) of 59 icteric and 55 non-icteric and in serum samples (**B**) of 28 icteric and 40 non-icteric BALB/c mice after RRV infection within 24 h of birth. The difference of caspase activity in the liver tissues between both groups is statistically significant (*) at day 8 (*p* < 0.001) and day 11 (*p* = 0.001) and in serum at day 5 (*p* = 0.015), 8 (*p* = 0.016) and 11 (*p* < 0.001). A positive correlation between caspase activity in liver and serum (**C**) (Pearson’s correlation coefficient 0.687, *p* < 0.001) shows that caspase activity in serum mirrors apoptosis of the liver in experimental BA mouse model (*p* < 0.001).

We then investigated whether the apoptotic liver injury of BA mice is reflected by increased caspase activity in the serum. For this purpose, we analyzed the caspase-3/-7 activity in the sera of icteric RRV+-BALB/c mice compared to non-icteric mice with a luminometric substrate assay, as used for the detection of caspase activity in the liver tissue extracts [24]. Initial experiments showed that the saline-treated control mice (*n* = 18) revealed similar caspase activity in the serum compared to the virus-treated non-icteric control mice (*n* = 22), indicating that cholestasis triggers caspase activation and apoptotic liver injury in BA mice (Supporting Information Figure S4). However, we found significantly (*p* < 0.05) higher caspase activity in the serum of the icteric mice (*n* = 28) at day 5, 8 and 11 compared to the non-icteric mice (*n* = 40) (Figure 3B). Moreover, the caspase activity in the serum of the RRV+-BALB/c mice was significantly (*p* < 0.01) correlated (r = 0.69) with the caspase activity in the liver tissue of those mice (Figure 3C). In addition, we measured the caspase activity in the liver (*n* = 98) and serum (*n* = 46) samples of C57BL/6 mice, which developed cholestasis in less than 30% of cases. The icteric C57BL/6 mice also showed an elevated caspase activity in the liver and serum compared to the non-icteric C57BL/6 mice; however, a significant peak could only be observed for day 8 within the observation period from day 5 to day 15 following RRV-treatment (Figure S5A,B). Compared to the icteric C57BL/6 mice, the icteric BALB/c mice revealed significantly higher caspase activity in the liver tissue extracts at day 5, 8 and 11 following the RRV treatment (Figure S6). Thus, the differences in the immune response between both mouse strains might contribute to the various extent of caspase activation and liver injury observed in those cholestatic mice.

### 3.4. Apoptotic Liver Injury Is Increased in BA Patients and Associated with Elevated Caspase Activity in Serum

Previous studies in BA demonstrated increased TUNEL reactivity in liver biopsies, especially in the bile duct epithelia [19,20]. In the present study, we investigated the role of apoptotic liver injury in liver biopsies from BA patients. We found increased caspase-3 activation and DNA fragmentation in the biliary epithelial cells of the liver tissues from BA patients compared to healthy liver tissues (Figure 1D–F).

**Figure 4:**
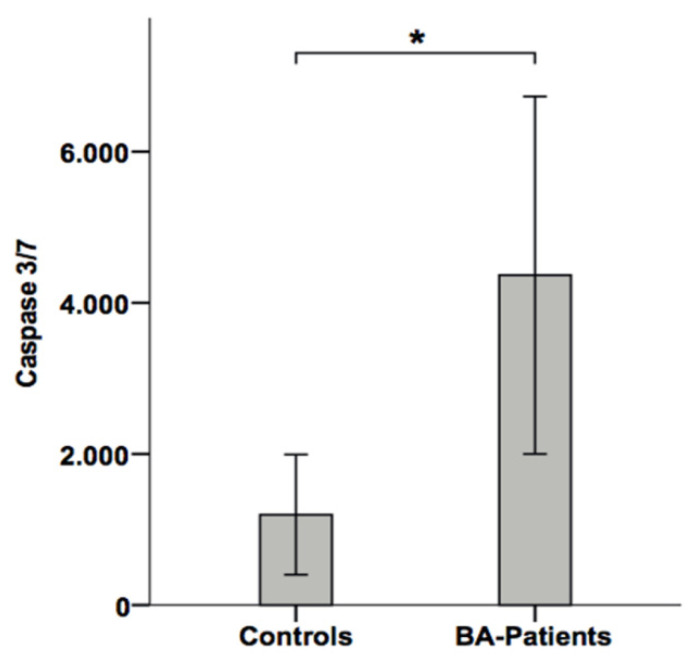
Comparing serum levels of effector caspases 3 and 7 in cholestatic neonates and healthy controls. A highly significant difference (*) (*p* < 0.01) was observed between BA patients and healthy or cholestatic control individuals.

**Figure 5:**
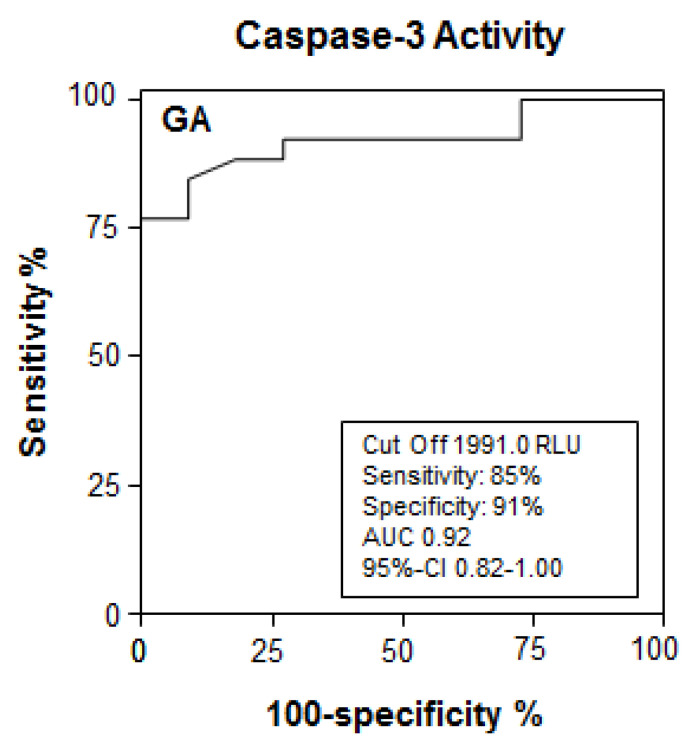
Receiver operating characteristics (ROC) analysis for detection of BA in children with cholestatic liver disease (C). The ROC analysis indicates the threshold for caspase-3/-7 activity with best compromise of sensitivity/specificity for detection of BA in pediatric patients with cholestasis.

We then investigated caspase-3/-7 activity in sera from BA patients by using the above mentioned luminometric substrate assay. BA patients revealed significantly increased caspase activity in sera compared to patients with other causes of cholestasis (Figure 4). To determine the discriminating value for caspase-3/-7 activity for a prediction of BA in cholestatic children, we performed an ROC analysis. As shown in Figure 5, a cut-off value of 1991 RLU of serum caspase-3/-7 activity correctly predicted BA in children with cholestasis with a sensitivity of 85% and a specificity of 91% (AUC 0.91). Thus, the serological detection of caspase activity revealed a very promising diagnostic performance for the detection of BA in cholestatic children.

## 4. Discussion

The diagnosis of BA is often delayed and treatment options, especially in progressed BA, remain limited [28]. Therefore, understanding the pathomechanism is necessary in order to identify novel biomarkers for early diagnosis and new therapeutic targets of this disease. There is rising evidence suggesting that increased apoptosis plays an important role in the pathogenesis of BA, leading to the regression and atrophy of bile ducts [19,23]. 

In this study we investigated the role of apoptosis in an experimental murine BA model and in human BA. We used an infective BA mouse model in which the RRV infection of newborn BALB/c mice results in histomorphological bile duct changes in approximately 70% of the rotavirus-infected pubs resembling human BA [29]. Previous studies from the murine model of BA have shown a time restricted gene upregulation of specific proteases, such as granzyme A and caspases, that triggers apoptosis induction with a peak at day 7 following RRV challenge [21,22]. The Cincinnati group demonstrated that apoptosis is induced in both murine intra- and extra-hepatic bile ducts, and depends on the synergistic effect of IFNγ and TNFα [11].

In the present study, we demonstrated increased caspase-3 activation and apoptosis (TUNEL reactivity) in the liver parenchyma and bile ducts of cholestatic RRV-infected newborn mice compared to non-cholestatic control mice. The subsequent analysis of caspase activity in the tissue extracts revealed a peak at day 8, followed by a continuous decline until day 15, which is contradictory to former theories considering ongoing apoptosis as a pathomechanism in experimental BA. Interestingly, in our study, the virus load also peaked at day 8 and then declined. Viral clearance has been associated with an increased number of CD3^+^ CTLs (27), which secrete TNF-α and express high levels of the CD95 ligand, both cytokines that trigger apoptosis. In addition, caspases can be activated by granzyme B, which is also produced from CTLs [30,31]. Whether caspase activation in experimental BA reflects the clearance of virus-infected cells and/or the apoptosis of non-infected liver cells remains nebulous.

Intriguingly, compared to the course of caspase activity in liver tissues, we found a very similar course of caspase-3/-7 activity in the sera of BA mice. The monitoring of caspase activity in the serum obviously mirrors apoptotic liver injury in experimental BA. Since no biomarker exists for the early detection and monitoring of disease activity in BA patients thus far, we evaluated caspase-3/-7 activity as possible biomarkers for the detection of BA. Similar to our findings in the BA mouse model, we found increased caspase-3 activation and apoptosis (TUNEL reactivity) in the liver tissues of BA patients compared to healthy liver tissues. Compared to murine BA tissues, in human BA, apoptosis was more restricted to the biliary epithelial cells. Those differences mainly show the limitations of translational research in biliary atresia. As recently discussed, the murine model of BA was used in a multitude of studies to investigate the mechanistic aspects of the disease. However, in the RRV-BALB/c model, pups decease within 21 days of life and, in our cohort, were sacrificed at day 15 of life [32]. In this short life span, the extent and the localization of injury can differ [32]. In human BA patients, epithelial cells acquire mesenchymal phenotypes and are, therefore, contributors to the process of apoptosis and fibrosis. In contrast, severe liver fibrosis has not yet been observed in the murine model of BA [33].

Patients with BA showed increased levels of caspase-3/-7 activity in the serum, compared to healthy control individuals. Moreover, BA patients revealed significantly higher caspase-3/-7 activity in sera compared to patients with other causes of cholestasis. Recently, it has been demonstrated that a decreased T-regulatory (Treg) cell number in the liver was associated with increased bile duct injury in experimental BA [34]. Similarly, in infants with BA, a decreased number of Treg cells has been associated with bile duct damage [3,35]. We have recently shown that the apoptosis of Treg cells in patients with chronic inflammatory disease is mirrored by an elevated caspase-3/-7 activity in the blood [36]. It might, therefore, be possible that the increased serum levels of caspase activity observed in BA patients at least partially reflects Treg cell apoptosis. In such a scenario, the increased apoptosis of Treg cells would result in a decreased Treg cell number, leading to impaired control of the immune response, increased inflammation and bile duct injury. A further intriguing finding of our study is the promising diagnostic performance (AUC value 0.91) of serological detection of caspase-3/-7 activity, which allows for the identification of BA in children with cholestasis with a high sensitivity and specificity.

The main limitation of the study is the missing follow up data for the human BA cohort and the lack of caspase-3/-7 dynamics in the infants. Therefore, the conclusions are based on the presented data of caspase-3/-7 activity in the human BA cohort at the time of the Kasai procedure and the murine RRV-BALB/c model.

In summary, the data of our study implicate that serological detection of caspase activation represents a suitable non-invasive biomarker for early identification of infants with BA and, moreover, distinguishes them from children with other cholestatic liver diseases. This is of clinical relevance since the restoration of the bile flow in BA patients should be performed as early as possible to prevent liver disease progression and the development of liver cirrhosis with associated complications. Furthermore, our observation that activated caspases play a major role in bile duct injury might open up new therapeutic strategies that are urgently needed for BA. In this respect, caspase-inhibitors, which have recently been demonstrated to attenuate liver injury and fibrosis in bile-duct ligated mice, could be promising agents for BA treatment [37]. Further studies in larger patient cohorts are warranted to evaluate the diagnostic performance of apoptosis biomarkers in BA patients.

## Figures and Tables

**Table 1:** Incidence of biliary atresia and recovering pups in RRV-infected BALB/c and C57BL/6 mice.

Mouse Strain (N)	Healthy (%)	Biliary Atresia (%)
BALB/c (N = 91)	28 (31%)	63 (69%)
C57BL/6 (N = 101)	79 (78%)	22 (22%)
Total	107	85

## Supplementary Materials

The following are available online at https://www.mdpi.com/article/10.3390/jcm10122718/s1.

## Abbreviations

BA	Biliary Atresia
LTx	Liver Transplantation
RRV	Rhesus Rotavirus
CK	Cytokeratin
IFNγ	Interferon-Gamma
TNFα	Tumor Necrosis Factor-alpha
Pfu	Plaque Forming Units
ELISA	Enzyme-Linked Immunosorbent Assay
H.E.	Hematoxylin and Eosin
PCR	Polymerase Chain Reaction
GAPDH	Glyceraldehyde-3-Phosphate-Dehydrogenase
TUNEL	Terminal Deoxynucleotidyl Transferase-mediated Deoxyuridine Triphosphated Nick End-Labelling
FFPE	Formalin-Fixed, Paraffin Embedded
SEM	Standard Error of Mean
DAB	Diaminobenzidin
RLU	Relative Light Units
CTL	Cytotoxic T Cell
