# Supplementary material for: Increased Serum Levels of Activated Caspases in Murine and Human Biliary Atresia
Source: J Clin Med. 2021 Jun 19;10(12):2718. doi: 10.3390/jcm10122718 (PMC8234421; doi:10.3390/jcm10122718)
Supplement: Supplementary file 1 [file jcm-10-02718-s001.zip › jcm-1206740-supplementary.pdf]

Supplementary material

rotavirus VP6
forward: 5′- GCGGTAGCGGTGTTATTTCC -3′
reverse: 5′- TTGTTTTGCTTGCGTCGG - 3′
GAPDH
forward: 5′- TGGTTAGACAATGAATACGGCTAC
reverse: 5′- GGTGGGTGGTCCAAGGTTTC

Figure S1. Primers used for PCR amplifications of the VP6 of RRV and GAPDH as the housekeeping gene of the liver.

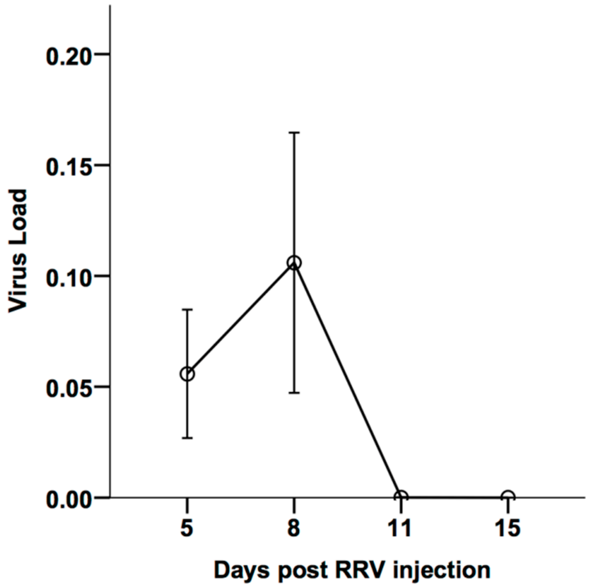

Figure S2. Replication of RRV in the liver from 22 BALB/c and 15 C57BL/6 mice showed a peak at day 8 after intraperitonal application. Quantitative real-time PCR of virus VP6 (* in relation to the housekeeping gene GAPDH; mean ± SEM) was performed at day 5, 8, 11 and 15 and revealed that the murine liver was cleared from the virus within two weeks after infection.

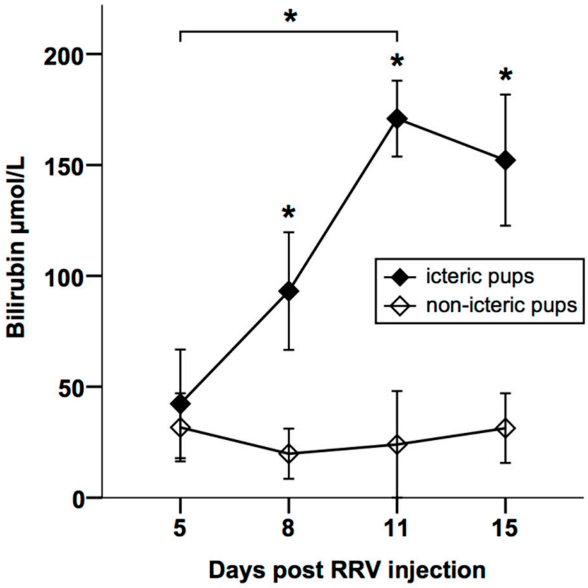

Figure S3. Bilirubin was tested in serum of 42 cholestatic and 13 non-icteric BALB/c mice. A significant (*) difference was seen at days 8 ($p = 0.031$), 11 ($p = 0.009$) and 15 ($p = 0.003$). A significant increase of bilirubin was observed between day 5 and day 11 ($p = 0.024$).

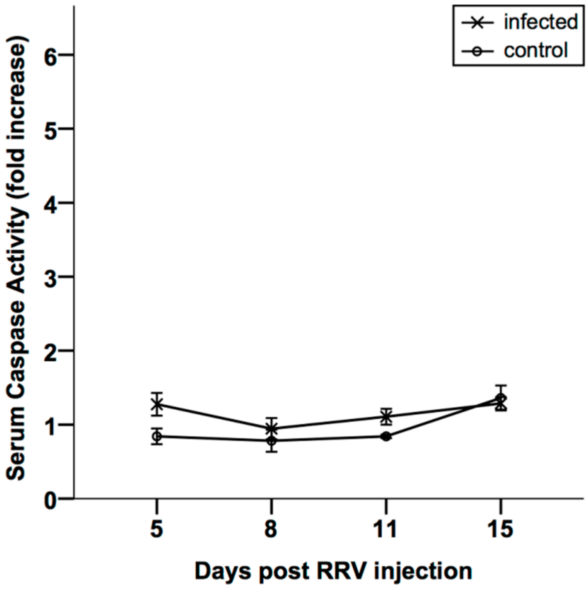

Figure S4. Caspase activity in serum (measured as fold increase compared to untreated controls) was tested in 22 RRV infected BALB/c mice, which remained unicteric and 18 non-infected controls. No difference could be observed at any of four time points, which demonstrates that RRV infection without clinical signs of cholestasis is not associated with caspase activity in serum of the pups.

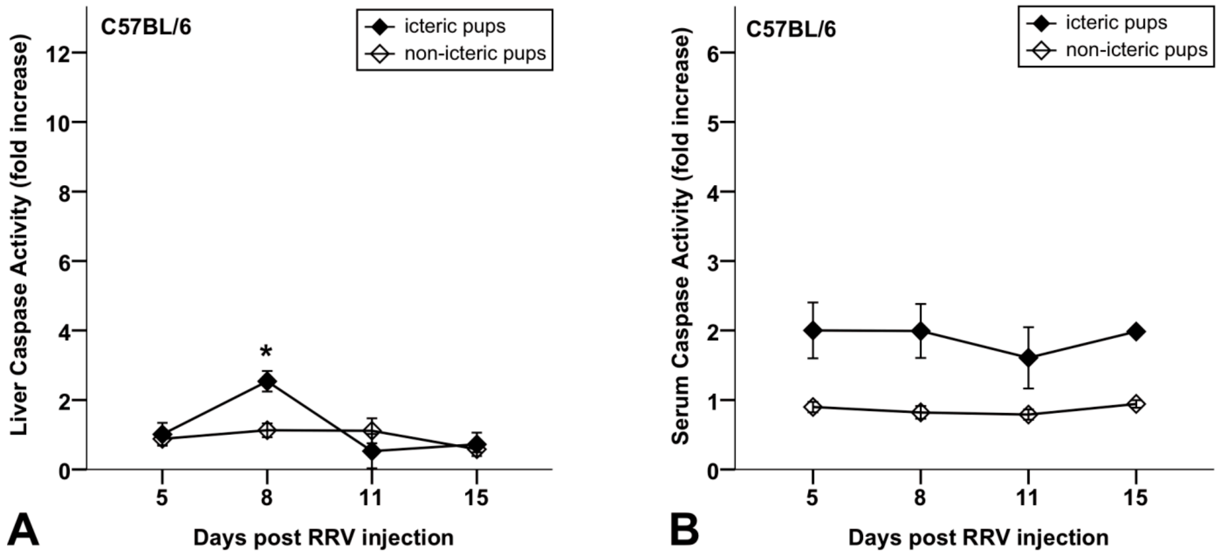

Figure S5. Activity of caspase-3 and caspase-7 measured by luminescent substrate assay (as fold increase compared to untreated controls; mean ± SEM) in liver of 98 icteric and non-icteric (**A**) and in serum samples of 46 icteric and non-icteric (**B**) C57BL/6 mice after RRV infection. A significant (*) difference of caspase activity between both groups was seen in the liver only at day 8 ($p = 0.002$) and in total in the serum ($p < 0.001$).

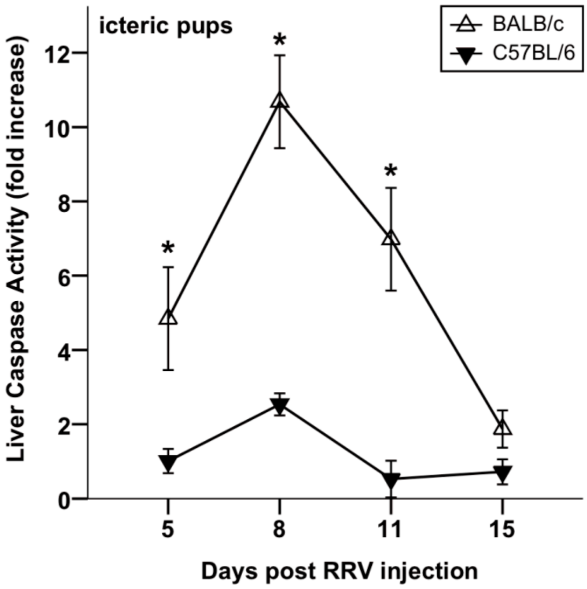

Figure S6. Icteric BALB/c mice showed a higher level of caspase activity (fold increase compared to untreated controls; mean ± SEM) in the liver than in C57BL/6 mice. Significance (*) was given at days 5, 8 and 11. (day 5: $p = 0.017$; day 8: $p < 0.001$; day 11: $p = 0.001$).
